# Supplementary material for: Perceived sensorimotor synchrony enhances pain modulation and attenuates laser-evoked potentials
Source: Commun Biol. 2025 Nov 25;8:1674. doi: 10.1038/s42003-025-09076-7 (PMC12647666; doi:10.1038/s42003-025-09076-7)
Supplement: Supplementary file 1 — Supplementary Information [file 42003_2025_9076_MOESM1_ESM.pdf]

Supplementary Information

Perceived sensorimotor synchrony enhances pain modulation and attenuates laser-evoked potentials

Xinyu Pan[1,2], Yian Xiao[1,2], Li Hu[1,2], Xuejing Lu[1,2*]

[1]State Key Laboratory of Cognitive Science and Mental Health, Institute of Psychology, Chinese Academy of Sciences, Beijing, China

[2]Department of Psychology, University of Chinese Academy of Sciences, Beijing, China

***Corresponding author**

E-mail: luxj@psych.ac.cn

Supplementary Experiment

The effects of perceived in-phase sensorimotor synchronization on spontaneous brain oscillations

This supplementary experiment was designed to explore the effects of perceived sensorimotor synchronization on spontaneous brain oscillations. As reported in the main manuscript, perceived sensorimotor synchrony significantly modulates pain perception at both behavioral and evoked neural response levels (Experiments 1-3). To further understand the neurophysiological mechanisms involved, we examined whether in-phase sensorimotor synchronization influence spontaneous brain oscillations.

Previous studies have shown that either engagement in rhythmic activities or increased cognitive involvement can influence spontaneous brain oscillations. For example, α-band activity over occipital regions is often linked to sensory inhibition and attentional disengagement[1], while γ-band oscillations are associated with sustained attention and immersive cognitive states[2,3]. Furthermore, the aperiodic exponent, reflecting the excitation/inhibition (E/I) balance in cortical networks, serves as a valuable indicator of neural dynamics[4]. In the main manuscript, we mainly focused on rapid nociceptive responses measured by event-related potentials. However, the analysis of spontaneous oscillations may yield further understanding of how sensorimotor synchronization regulates cognitive and affective processes involved in pain modulation.

To this end, this experiment aimed to assess changes in oscillatory power and aperiodic exponents following a synchronized sensorimotor task compared to a rest condition. We hypothesized that perceived in-phase sensorimotor synchronization would modulate spontaneous brain oscillations differently from the rest condition.

Methods

Participants

Twenty participants were recruited (13 females and 7 males, mean ± SD age = 22.3 ± 2.6 years, age range: 19 - 29 years). The sample size was determined through *a prior* power analysis for

one-way repeated-measures ANOVA, focusing on the within-factor effects across three groups. The analysis indicated a minimum required sample size of 12 to achieve adequate statistical power. To account for potential data loss and variability, we recruited 20 participants.

Stimuli, equipment, experimental design, and procedures

The auditory stimuli and drumming equipment used in this supplementary experiment were identical to those described in Experiments 1-3 of the main manuscript. As shown in Supplementary Fig. 3, this experiment employed a within-subjects design, where each participant underwent three blocked conditions: 90-bpm in-phase synchrony, 120-bpm in-phase synchrony, and a rest condition. Each condition lasted for 5 minutes. In the two sensorimotor synchronization conditions (90-bpm and 120-bpm), participants were instructed to synchronize their drumming strokes to the demonstrated tempo guided by aligned auditory and visual cues (as per the in-phase synchrony condition in Experiment 2 of the main manuscript). In the rest condition, participants were asked to remain relaxed without falling asleep. Open-eyed resting-state EEG (rsEEG) data were collected for 1 minute before and after each condition (i.e., the pre-rsEEG and the post-rsEEG sessions). To minimize potential carryover effects between the two sensorimotor synchronization blocks, the presentation order of the 90-bpm and 120-bpm conditions was counterbalanced across participants, and a rest condition was inserted between them.

EEG recording and preprocessing

EEG data acquisition followed the same protocol as Experiment 3 in the main manuscript. For preprocessing, continuous EEG data were first bandpass filtered from 1 to 100 Hz, with a notch filter applied at 49-51 Hz to remove electrical noise. Subsequently, rsEEG data were segmented into 2-second epochs. EEG epochs were visually inspected, but no electrodes required interpolation or trials were excluded. Trials contaminated by eye blinks, eye movements, and movements were corrected using *runica* algorithm. Finally, EEG data were re-referenced to the average of the bilaterial mastoids.

Frequency domain analysis

Power spectral density (PSD) of EEG signals was calculated using the Welch's method. This

operation yielded an EEG power spectrum for each participant and condition. Then, we divided the power spectra into five canonical frequency bands (δ: 1-3 Hz; θ: 4-7 Hz; α: 8-12 Hz; β: 13-29 Hz; low γ: 30-60 Hz; and high γ: 61-90 Hz)[5-7]. Total power within each frequency band was computed as the mean power spectra of the respective frequency range at each electrode. Group-level power spectra were obtained by averaging individual data, and scalp topographies for each frequency band were computed by spline interpolation.

In addition, PSD were decomposed into periodic and aperiodic components using the Fitting Oscillations and One-Over-F (FOOOF) algorithm[4], with the following settings: aperiodic mode = "fixed"; peak width limits = [1, 8]; maximum number of peaks = 8, minimum peak height = 0.05. Aperiodic exponent values were extracted for each participant and each electrode, as the aperiodic exponent (i.e., the slope of aperiodic component) has been considered as a measure of the E/I balance[8]. Specifically, a higher exponent (steeper spectra) indicates a shift toward inhibition, while a lower exponent suggests a shift toward excitation[9]. Group-level aperiodic exponents were obtained by averaging across participants for each session, and scalp topographies were computed by spline interpolation.

Statistical analysis

For rsEEG analyses in this supplementary experiment, repeated-measures one-way ANOVAs were performed at each electrode to examine changes in PSD of each frequency band and aperiodic exponent between pre- and post-rsEEG sessions. To control the Family-Wise Error Rate (FWER) across all multiple comparisons among electrodes, the Holm-Bonferroni correction was applied. Subsequently, for electrodes clusters that remained significant after Holm-Bonferroni correction, post-hoc tests were conducted to clarify the results of pairwise comparisons.

Results

Spontaneous brain oscillations

Supplementary Fig. 4A shows the rsEEG spectra of spontaneous brain oscillations for three conditions. After Holm-Bonferroni correction for multiple comparisons across all electrodes, significant effects of condition were found on changes in PSD within the α-band (observed

broadly across electrodes over parieto-occipital area; $F_{(2,19)} = 10.99$, $P < 0.001$, $\eta_p^2 = 0.37$) and the high γ-band (observed broadly across electrodes over the centro-parieto-occipital area; $F_{(2,19)} = 9.00$, $P = 0.001$, $\eta_p^2 = 0.32$).

We then performed post-hoc pairwise comparisons on the average spectral power across the significant electrodes within these bands. These comparisons showed that α-band spectral power was significantly decreased after the rest condition compared to the two sensorimotor synchronization conditions (90-bpm vs. rest: $P = 0.006$; 120-bpm vs. rest: $P = 0.002$), while high γ-band spectral power was significantly increased after the two sensorimotor synchronization conditions than the rest condition (90-bpm vs. rest: $P = 0.017$; 120-bpm vs. rest: $P = 0.001$; Supplementary Fig. 4B). Notably, the observed effect of high γ oscillations are unlikely to be attributed to muscle artifacts, as γ oscillations associated with muscular contractions are typically distributed over peripheral electrode sites[10]. No significant difference across conditions was observed in the δ, θ, β, or low γ frequency bands (all $P > 0.05$, see Supplementary Fig. 5).

Aperiodic exponent values

Furthermore, as shown in Supplementary Fig. 4C and 4D, a significant main effect of condition was found for changes in aperiodic exponent (observed broadly across electrodes over the centro-parieto-occipital area; $F_{(2,19)} = 8.28$, $P = 0.003$, $\eta_p^2 = 0.48$). Post-hoc pairwise comparisons revealed that the aperiodic exponent was significantly decreased after the two sensorimotor synchronization conditions compared to the rest condition (90-bpm vs. rest: $P = 0.026$; 120-bpm vs. rest: $P = 0.002$). No differences between 90-bpm and 120-bpm conditions were found in aperiodic exponent.

Discussion

This supplementary experiment investigated the effects of perceived in-phase synchrony on spontaneous brain oscillations, extending the pain modulation findings reported in the main manuscript. The results reveal significant modulations in α-band activity, high γ-band oscillations, and aperiodic exponent. However, there are no significant differences observed between the 90-bpm and 120-bpm conditions, suggesting that at least within this tempo range

sensorimotor synchronization exerts a consistent influence on spontaneous brain oscillations.

Although a recent study suggested that 120 bpm may be closer to the individual preferred tempo for many people[11], our results did not detect significant differences in spontaneous brain oscillations between these two tempos. The comparable effects at 90-bpm and 120-bpm may be attributed to the fact that both tempos fall within the empirically established optimal range for human sensorimotor synchronization (approximately 83–234 bpm), which facilitates comfortable and natural rhythmic movement[12,13]. Furthermore, as suggested by Yi et al.[11], the alignment between an external rhythm and an individual's internal rhythmic tendency (e.g., spontaneous production rate) may be a more critical for optimal response than the absolute tempo. It is possible that both 90 bpm and 120 bpm aligned adequately with the intrinsic rhythms of a substantial portion of our sample, leading to comparable modulation of brain oscillations.

Specifically, we observed that in-phase synchronization was associated with increased high γ-band oscillations and a significant reduction in the aperiodic exponent over centro-parieto-occipital regions, when compared with the rest condition. Increased γ-band oscillation is frequently linked to heightened cognitive engagement and immersive experiences[2,14,15]. This interpretation is consistent with evidence from meditation and mindfulness studies, which reports increased γ-band activity in advanced practitioners of various meditation practices[16,17]. Furthermore, a recent study has linked increased γ-band oscillations in centro-parieto-occipital areas to pain relief and immersion[3]. The observed decrease in the aperiodic exponent further suggests a shift toward a excitatory state in these areas[4,8], a change associated with heightened embodiment that may promote sensorimotor integration and reduce pain perception[18].

Furthermore, we also observed maintained α-band oscillations in parieto-occipital areas following two in-phase synchrony conditions, when compared to the rest condition. Although α-band activity is typically linked to relaxed wakefulness and sensory disengagement[1], the observed effect might reflect reduced visual processing or disengagement from visual input during the drumming-to-music task[19,20], potentially limiting competing sensory inputs and contributing to analgesic effects.

These findings should be interpreted in light of several limitations. First, the absence of a control condition with asynchrony movement prevents definitive conclusions regarding whether the observed effects are specific to perceived synchrony or simply reflect general rhythmic motor activity or arousal. Although we interpret these results in the context of synchrony, the lack of an appropriate control mandates caution. Second, the absence of trial-wise subjective measures limits mechanistic interpretation. Inferences regarding cognitive engagement and immersion are based on oscillatory correlates from the literature[2,14,15], rather than direct psychometric assessments. Moreover, the lack of continuous pain rating during this experiment precludes direct correlation between neural changes and subjective pain experience. Thus, while these oscillations represent promising candidate mechanisms, future studies should incorporate real-time subjective measures to establish precise brain–behavior relationship. Third, the fixed placement of the rest condition between two in-phase synchrony conditions may introduce order effects such as fatigue or habituation. Synchronization conditions were counterbalanced though. Future designs should consider full condition randomization or matched control tasks to improve baseline comparisons.

Given these limitations, future studies would benefit from including active asynchrony control conditions, integrating validated psychometric measures of immersion and related perceptual state.

References

1. Klimesch, W. Alpha-band oscillations, attention, and controlled access to stored information. *Trends Cogn. Sci*. **16**, 606-617 (2012).
2. Clayton, M. S., Yeung, N. & Kadosh, R. C. The roles of cortical oscillations in sustained attention. *Trends Cogn. Sci.* **19**, 188-195 (2015).
3. Li, J. *et al*. The analgesic effects and neural oscillatory mechanisms of virtual reality scenes based on distraction and mindfulness strategies in human volunteers. *Br. J. Anaesth.* **131**, 1082-1092 (2023).
4. Donoghue, T. *et al*. Parameterizing neural power spectra into periodic and aperiodic components. *Nat. Neurosci*. **23**, 1655-1665 (2020).
5. Buzsaki, G. & Draguhn, A. Neuronal oscillations in cortical networks. *Science* **304**, 1926-1929 (2004).
6. Başar, E., Başar-Eroglu, C., Karakaş, S. & Schürmann, M. Gamma, alpha, delta, and theta oscillations govern cognitive processes. *Int. J. Psychophysiol.* **39**, 241-248 (2001).
7. Keil, A. *et al.* Recommendations and publication guidelines for studies using frequency

domain and time-frequency domain analyses of neural time series. *Psychophysiology* **59**, e14052 (2022).

8. Gao, R., Peterson, E. J. & Voytek, B. Inferring synaptic excitation/inhibition balance from field potentials. *NeuroImage* **158**, 70-78 (2017).
9. Lendner, J. D. *et al.* An electrophysiological marker of arousal level in humans. *Elife* **9**, e55092 (2020).
10. Muthukumaraswamy, S. D. High-frequency brain activity and muscle artifacts in MEG/EEG: a review and recommendations. *Front. Hum. Neurosci.* **7**, 138 (2013).
11. Yi, W., Palmer, C., Serian, A. & Roy, M. Individualizing musical tempo to spontaneous rates maximizes music-induced hypoalgesia. *Pain* **166**, 1761-1768 (2025).
12. Repp, B. H. & Su, Y. H. Sensorimotor synchronization: a review of recent research (2006-2012). *Psychon. Bull. Rev.* **20**, 403-452 (2013).
13. Van Noorden, L. & Moelants, D. Resonance in the perception of musical pulse. *J. New Music Res.* **28**, 43-66 (1999).
14. Engel, A. K., Fries, P. & Singer, W. Dynamic predictions: oscillations and synchrony in top–down processing. *Nat. Rev. Neurosci.* **2**, 704-716 (2001).
15. Škola, F. & Liarokapis, F. Examining the effect of body ownership in immersive virtual and augmented reality environments. *Vis. Comput.* **32**, 761-770 (2016).
16. Cahn, B. R., Delorme, A. & Polich, J. Occipital gamma activation during Vipassana meditation. *Cogn. Process* **11**, 39-56 (2010).
17. Lutz, A., Greischar, L. L., Rawlings, N. B., Ricard, M. & Davidson, R. J. Long-term meditators self-induce high-amplitude gamma synchrony during mental practice. *Proc. Natl. Acad. Sci. U.S.A.* **101**, 16369-16373 (2004).
18. Montull, L., Vázquez, P., Rocas, L., Hristovski, R. & Balagué, N. Flow as an embodied state. Informed awareness of slackline walking. *Front. Psychol.* **10**, 2993 (2020).
19. Jensen, O., Gelfand, J., Kounios, J. & Lisman, J. E. Oscillations in the alpha band (9–12 Hz) increase with memory load during retention in a short-term memory task. *Cereb. Cortex* **12**, 877-882 (2002).
20. Iurilli, G. *et al.* Sound-driven synaptic inhibition in primary visual cortex. *Neuron* **73**, 814-828 (2012).

Supplementary Figures

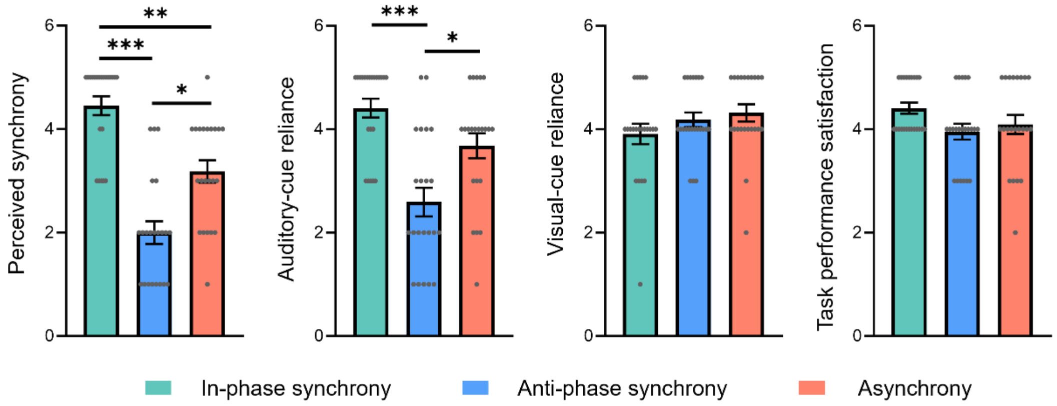

Supplementary Fig. 1 Manipulation check (n = 22 for each group; total N = 66). Participants' ratings of perceived audio-visual synchrony (Q1), auditory-cue reliance (Q2), visual-cue reliance (Q3), and subjective task performance (Q4) across three conditions: in-phase synchrony (green), anti-phase synchrony (blue), and asynchrony (red). Error bar represents ± one standard error of the mean; *** $P < 0.001$; ** $P < 0.01$; * $P < 0.05$.

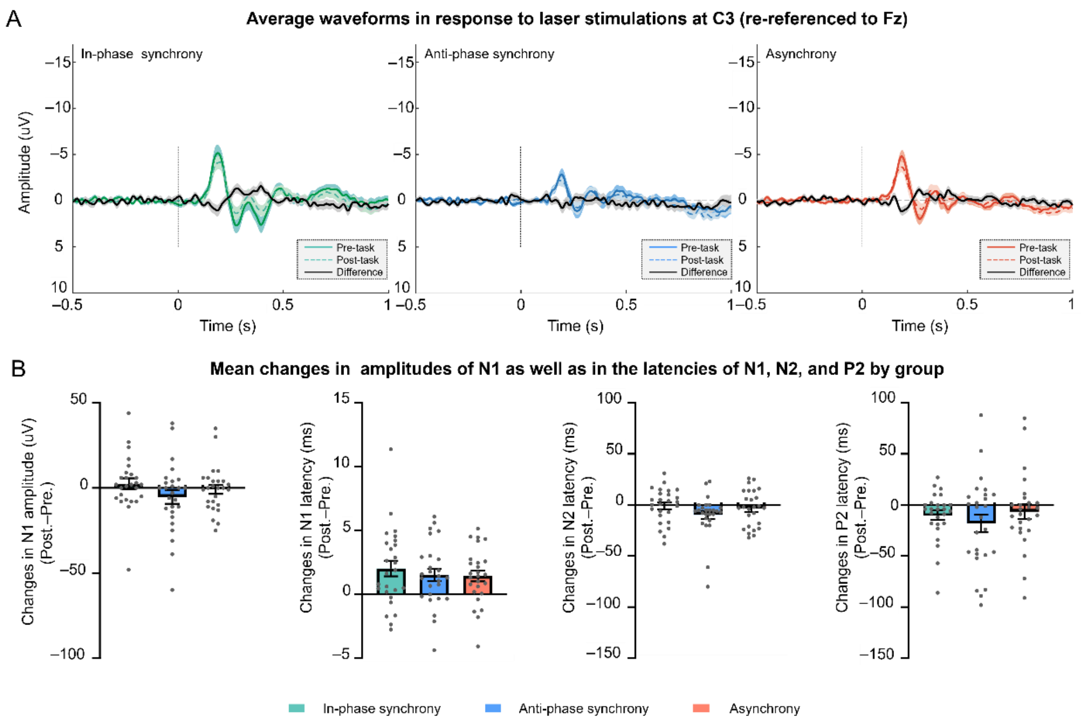

Supplementary Fig. 2 Changes in laser-evoked N1 amplitude and N1, N2, P2 latencies by groups (n = 26 for each group; total N = 78). **A** Average waveforms at C3 electrode (with Fz as the reference electrode) of each group in response to laser stimulations in the pre-task (solid line) and the post-task (dash line) sessions (black lines were difference waves between two sessions). **B** Comparisons of changes in N1, N2, and P2 latencies and N1 amplitude among in-phase synchrony (green), anti-phase synchrony (blue), and asynchrony (red) groups. Error bar represents ± one standard error of the mean. Pre.: the pre-task session; Post.: the post-task session.

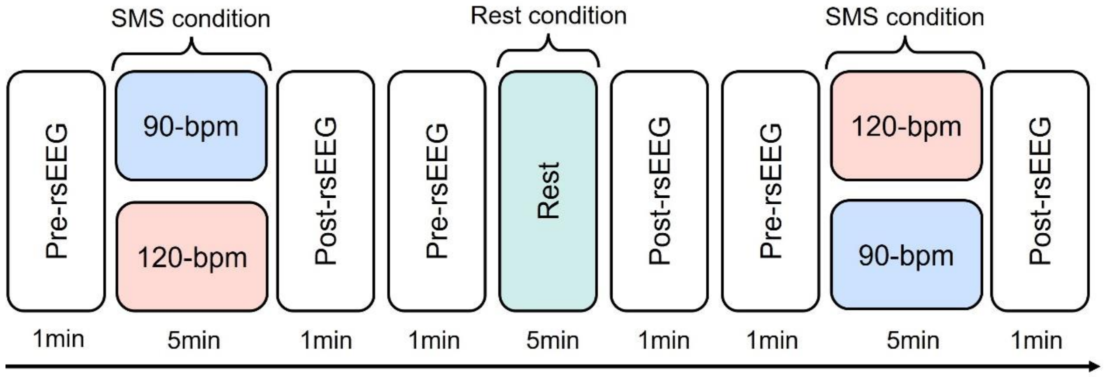

Supplementary Fig. 3 The design of Supplementary Experiment. SMS: sensorimotor synchronization; rsEEG: resting-state EEG; bpm: beat per minute.

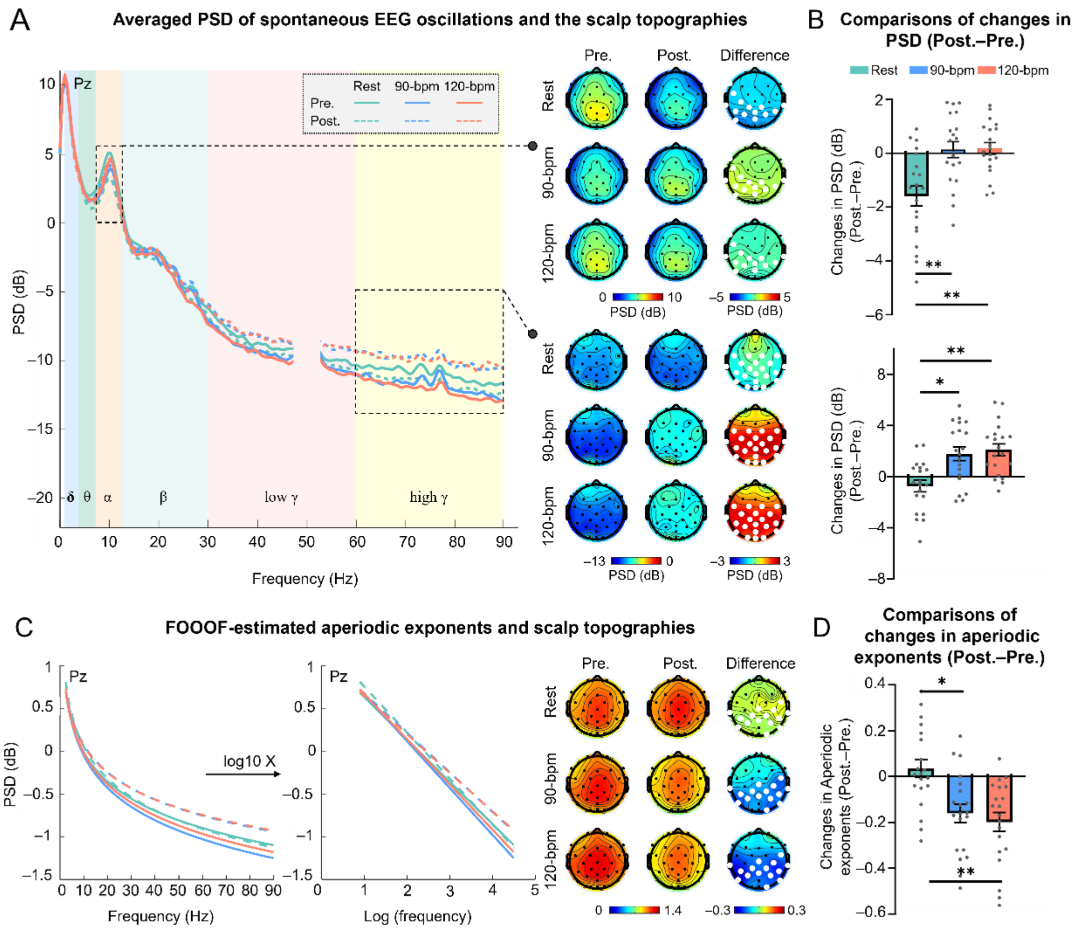

Supplementary Fig. 4 Results of Supplementary Experiment (n = 20). **A** Averaged power spectral density (PSD) of spontaneous EEG oscillations at Pz electrode in the pre-task (solid line) and post-task (dash line) sessions for the rest (green), 90-bpm (blue), and 120-bpm (red) conditions, as well as the scalp topographies. Electrode sites with statistically significant group differences after Holm-Bonferroni correction were marked with white dots. **B** Comparisons of changes in PSD of α- and high γ-band oscillations within the significant electrode clusters. **C** A demonstration of aperiodic exponents estimated at Pz electrode using the FOOOF algorithm for each session and condition, as well as the scalp topographies. **D** Comparisons of changes in aperiodic exponents for each condition within the significant electrode clusters. Error bar represents ± one standard error of the mean. Pre.: the pre-rsEEG session; Post.: the post-rsEEG session; bpm: beat per minute. Pre.: the pre-task session; Post.: the post-task session; bpm: beat per minute; ** $P < 0.01$; * $P < 0.05$.

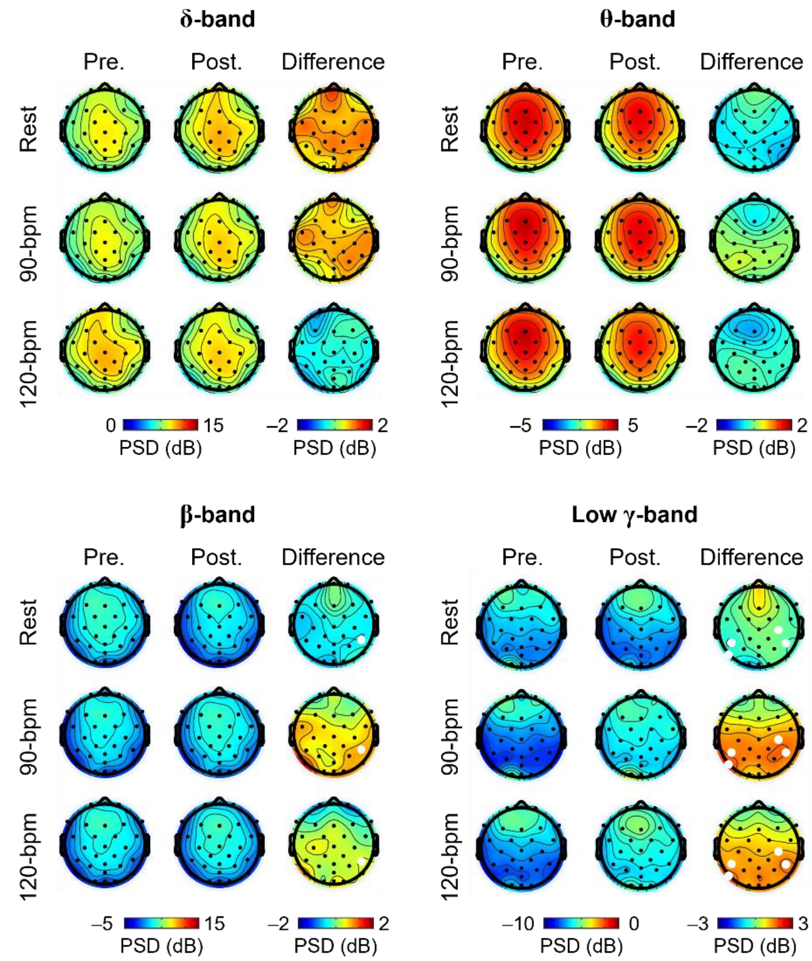

Supplementary Fig. 5 The scalp topographies of δ-, θ-, β- and low γ-band oscillations in the pre-task and post-task sessions for the rest, 90-bpm, and 120-bpm conditions. While significant effect of condition was observed at a few isolated electrodes within the β and low γ-bands (marked with white dots), no significant clusters of spatially adjacent electrodes were identified for these frequency bands. Pre.: the pre-rsEEG session; Post.: the post-rsEEG session; PSD: power spectral density; bpm: beat per minute.
