# Supplementary material for: Perceived sensorimotor synchrony enhances pain modulation and attenuates laser-evoked potentials
Source: Commun Biol. 2025 Nov 25;8:1674. doi: 10.1038/s42003-025-09076-7 (PMC12647666; doi:10.1038/s42003-025-09076-7)
Supplement: Supplementary file 2 — Description of Additional Supplementary Materials [file 42003_2025_9076_MOESM2_ESM.pdf]

Description of Additional Supplementary Files

File name: Supplementary Data 1

Description: the numerical source data underlying the graphs
