# Supplementary material for: Perceived sensorimotor synchrony enhances pain modulation and attenuates laser-evoked potentials
Source: Commun Biol. 2025 Nov 25;8:1674. doi: 10.1038/s42003-025-09076-7 (PMC12647666; doi:10.1038/s42003-025-09076-7)
Supplement: Supplementary file 4 — Reporting summary [file 42003_2025_9076_MOESM4_ESM.pdf]

nature portfolio

Corresponding author(s): Xuejing Lu

Last updated by author(s): Oct 13, 2025

Reporting Summary

Nature Portfolio wishes to improve the reproducibility of the work that we publish. This form provides structure for consistency and transparency in reporting. For further information on Nature Portfolio policies, see our Editorial Policies and the Editorial Policy Checklist.

Statistics

For all statistical analyses, confirm that the following items are present in the figure legend, table legend, main text, or Methods section.

n/a	Confirmed	
☐	☒	The exact sample size (*n*) for each experimental group/condition, given as a discrete number and unit of measurement
☐	☒	A statement on whether measurements were taken from distinct samples or whether the same sample was measured repeatedly
☐	☒	The statistical test(s) used AND whether they are one- or two-sided<br>*Only common tests should be described solely by name; describe more complex techniques in the Methods section.*
☒	☐	A description of all covariates tested
☐	☒	A description of any assumptions or corrections, such as tests of normality and adjustment for multiple comparisons
☐	☒	A full description of the statistical parameters including central tendency (e.g. means) or other basic estimates (e.g. regression coefficient) AND variation (e.g. standard deviation) or associated estimates of uncertainty (e.g. confidence intervals)
☐	☒	For null hypothesis testing, the test statistic (e.g. *F*, *t*, *r*) with confidence intervals, effect sizes, degrees of freedom and *P* value noted<br>*Give P values as exact values whenever suitable.*
☒	☐	For Bayesian analysis, information on the choice of priors and Markov chain Monte Carlo settings
☒	☐	For hierarchical and complex designs, identification of the appropriate level for tests and full reporting of outcomes
☐	☒	Estimates of effect sizes (e.g. Cohen's *d*, Pearson's *r*), indicating how they were calculated

Our web collection on statistics for biologists contains articles on many of the points above.

Software and code

Policy information about availability of computer code

Data collection	The E-prime 3.0 software (Psychology Software Tools, Inc; Pittsburgh, USA) was used to presented the laser stimulation.
Data analysis	Behavioral data were analyzed using IBM SPSS Statistics (IBM Corp., Armonk, NY). EEG data were processed using EEGLAB(v2023.1), an open-source toolbox running under the MATLAB environment (The MathWorks, Inc., Natick, Massachusetts, USA). The EEG auto-analysis scripts are publicly available in the Open Science Foundation repository https://osf.io/5z24n/.

For manuscripts utilizing custom algorithms or software that are central to the research but not yet described in published literature, software must be made available to editors and reviewers. We strongly encourage code deposition in a community repository (e.g. GitHub). See the Nature Portfolio guidelines for submitting code & software for further information.

Data

Policy information about availability of data

All manuscripts must include a data availability statement. This statement should provide the following information, where applicable:

- Accession codes, unique identifiers, or web links for publicly available datasets
- A description of any restrictions on data availability
- For clinical datasets or third party data, please ensure that the statement adheres to our policy

The primary data are publicly available (https://osf.io/5z24n/).

Research involving human participants, their data, or biological material

Policy information about studies with human participants or human data. See also policy information about sex, gender (identity/presentation), and sexual orientation and race, ethnicity and racism.

Reporting on sex and gender	A total of 244 healthy participants (including the exploratory experiment described in Supplementary Experiment) were recruited for this study (139 females, mean age ± SEM = 23.2 ± 2.43 years, age range: 18 - 30 years).
Reporting on race, ethnicity, or other socially relevant groupings	We did not report the information about race, ethnicity, or other socially relevant groupings.
Population characteristics	None of the participants reported any medical conditions related to acute or chronic pain, cardiovascular or neurological diseases, psychiatric disorders, current use of medication, or any noticeable hand injuries.
Recruitment	Participants were recruited through advertisements posted on social media platforms such as WeChat, consisting of young adults aged 18-30 years, which may limit the generalizability of the findings to broader populations.
Ethics oversight	This research received approval from the Ethics Committee of the Institute of Psychology, Chinese Academy of Sciences, China (ID: H21103).

Note that full information on the approval of the study protocol must also be provided in the manuscript.

Field-specific reporting

Please select the one below that is the best fit for your research. If you are not sure, read the appropriate sections before making your selection.

☒ Life sciences ☐ Behavioural & social sciences ☐ Ecological, evolutionary & environmental sciences

For a reference copy of the document with all sections, see nature.com/documents/nr-reporting-summary-flat.pdf

Life sciences study design

All studies must disclose on these points even when the disclosure is negative.

Sample size	Sample size was determined using G*Power software (available at http://www.Gpower.hhu.de/en.html) with statistical power (1-β) set at 0.8, a large effect size (f = 0.4), and a significance level (α) at 0.05.
Data exclusions	No data were excluded.
Replication	No direct replication was conducted, but the directionality of results was consistent across experiments.
Randomization	In Experiments 1-3, all participants were randomly assigned to different experimental conditions; Supplementary Experiment employed a within-subject design where all participants underwent all conditions, thus requiring no randomization.
Blinding	Participants were kept unaware of the study's hypothesis regarding pain modulation. Additionally, the experimenter who administered the pain rating tasks was blinded to the experimental conditions to minimize potential bias.

Reporting for specific materials, systems and methods

We require information from authors about some types of materials, experimental systems and methods used in many studies. Here, indicate whether each material, system or method listed is relevant to your study. If you are not sure if a list item applies to your research, read the appropriate section before selecting a response.

Materials & experimental systems

n/a	Involved in the study
☒	☐ Antibodies
☒	☐ Eukaryotic cell lines
☒	☐ Palaeontology and archaeology
☒	☐ Animals and other organisms
☒	☐ Clinical data
☒	☐ Dual use research of concern
☒	☐ Plants

Methods

n/a	Involved in the study
☒	☐ ChIP-seq
☒	☐ Flow cytometry
☒	☐ MRI-based neuroimaging

Plants

Seed stocks	n/a
Novel plant genotypes	n/a
Authentication	n/a
